# labelSeg: segment annotation for tumor copy number alteration profiles

**DOI:** 10.1093/bib/bbad541

**Published:** 2024-01-31

**Authors:** Hangjia Zhao, Michael Baudis

**Affiliations:** Department of Molecular Life Sciences, University of Zurich, Winterthurerstrasse 190, 8057, Zurich, Switzerland; Computational Oncogenomics Group, Swiss Institute of Bioinformatics, Winterthurerstrasse 190, 8057, Zurich, Switzerland; Department of Molecular Life Sciences, University of Zurich, Winterthurerstrasse 190, 8057, Zurich, Switzerland; Computational Oncogenomics Group, Swiss Institute of Bioinformatics, Winterthurerstrasse 190, 8057, Zurich, Switzerland

**Keywords:** somatic copy number alterations, segment annotation, density-based clustering

## Abstract

Somatic copy number alterations (SCNAs) are a predominant type of oncogenomic alterations that affect a large proportion of the genome in the majority of cancer samples. Current technologies allow high-throughput measurement of such copy number aberrations, generating results consisting of frequently large sets of SCNA segments. However, the automated annotation and integration of such data are particularly challenging because the measured signals reflect biased, relative copy number ratios. In this study, we introduce *labelSeg*, an algorithm designed for rapid and accurate annotation of CNA segments, with the aim of enhancing the interpretation of tumor SCNA profiles. Leveraging density-based clustering and exploiting the length–amplitude relationships of SCNA, our algorithm proficiently identifies distinct relative copy number states from individual segment profiles. Its compatibility with most CNA measurement platforms makes it suitable for large-scale integrative data analysis. We confirmed its performance on both simulated and sample-derived data from The Cancer Genome Atlas reference dataset, and we demonstrated its utility in integrating heterogeneous segment profiles from different data sources and measurement platforms. Our comparative and integrative analysis revealed common SCNA patterns in cancer and protein-coding genes with a strong correlation between SCNA and messenger RNA expression, promoting the investigation into the role of SCNA in cancer development.

## INTRODUCTION

Genomic instability is a nearly ubiquitous hallmark of cancer. Cancer cells often lose the ability to maintain genome integrity, but the molecular basis of genomic instability is not always clear [1]. One consequence of genomic instability is the occurrence of somatic copy number alterations (SCNAs), which are changes in the copy number of chromosome segments from the regional allele count in somatic (i.e. post germline) tissues. SCNAs represent the by extent largest contributions to genomic variation in cancer, with genetic components affected by SCNA frequently conferring selective advantages to affected cells, thereby promoting cancer initiation and progression [2].

Various methods are employed to detect SCNAs, ranging from (molecular-)cytogenetic and locus-specific techniques such as karyotype analysis, interphase fluorescence in-situ hybridization (FISH) and spectral karyotyping (SKY) to genomic microarrays and next-generation sequencing (NGS) methods. However, achieving a comprehensive capture of all CNA information remains challenging with any individual approach, given the distinct detection biases and limitations inherent in different technologies and platforms. These differences manifest in various aspects, such as the upper and lower detection sensitivity for CNA events of differing sizes, the requirement for matched reference samples and the capability to detect allele-specific CNA events [3], and are compounded by varying processing pipelines.

In the meta-analysis of large and heterogeneous SCNA datasets, a major challenge lies in interpreting and comparing segmented copy number profiles derived from raw intensity data obtained using techniques such as microarrays and NGS. Frequently, such segmented CNA profiles constitute the solely accessible data due to privacy concerns related e.g. to the exposure of single nucleotide polymorphism (SNP) data. Typically, the available CNA data are represented through genomic segments with the relative abundance of the DNA expressed as the log R ratio ($\log $R), calculated by taking the $\log $2 of the ratio between the observed intensity of a sample and a reference intensity. Notably, it is a normalized metric, spotlighting changes in relative copy numbers rather than an absolute copy number at a given genomic location. However, such a representation leads to challenges when comparing SCNA profiles across datasets, which are further compounded by the fact that signal scale and noise levels can vary widely across samples due to variations in clonal sample purity, ploidy, experimental steps in bio-sample preparation, measurement platform and other factors.

To overcome the challenges of inter-sample comparisons, some tools such as VarScan2 [4], CODEX [5] and CNVkit [6] adopt fixed, empirical thresholds to classify segments with signals beyond these thresholds into ‘duplication’ or ‘deletion’ CNA categories. However, the selection of these thresholds can significantly impact the results of such analyses. Lower cut-off values improve the sensitivity of variant detection—especially for samples with admixed non-cancer tissue—but can lead to many false positive calls. In contrast, larger cut-off values may enhance the calling precision but risk overlooking true variants, thereby introducing systematic bias particularly due to cancer-specific differences in sample purity. Some studies [7, 8] optimize this process by incorporating purity estimation and adjusting thresholds based on the estimated purity. However, this added estimation complicates SCNA profiling by necessitating manual selection of the most suitable solutions [9]. Several complicated models have been developed to improve SCNA detection, including Gaussian mixture models [10, 11], hidden Markov models [12–14] or a combination of both [15]. Although theoretically promising for providing more accurate CNA calls, these models often require raw data from individual measurement platforms that may not be accessible, or allele-specific data which certain technologies lack. Furthermore, most of these methods are designed to provide an absolute copy number quantification, rather than addressing the identification of distinct empirical CNA types. These types, characterized not only by CNA amplitude but also by CNA length, include broad CNAs and focal CNAs. These types differ in size, magnitude and potential functional implications [16]. While GISTIC2 [17] can distinguish between these CNA types or levels, its primary objective is to identify recurrent amplified or deleted genomic regions across a set of samples, rather than accurately determining CNA levels in individual samples. Thus, the fast and accurate annotation of individual CNA segment profiles remains a challenging problem in the field of SCNA profiling.

To address the above challenges, we developed a novel method called *labelSeg*. This method not only accurately annotates individual CNA profiles but also scales seamlessly to accommodate large-scale studies encompassing numerous samples. By utilizing estimated calling thresholds from individual segment profiles, *labelSeg* identifies various levels of CNAs without requiring prior information such as purity estimation. Its one-dimensional clustering approach, coupled with a direct cut-off strategy using the estimated thresholds, enables rapid processing of CNA profiles. The only input required is copy number segment profiles, which can be generated by most CNA measurement platforms and processing pipelines. These attributes make *labelSeg* particularly well-suited for large-scale meta-analyses. In validation cohorts from The Cancer Genome Atlas (TCGA) [18] covering diverse cancer types, *labelSeg* showed superior performance compared with GISTIC2 and fixed thresholds. Moreover, through an integrative analysis spanning four research projects, involving >2000 glioblastoma samples and >1200 lung squamous cell carcinoma (LUSC) samples, *labelSeg* demonstrated its capability to achieve fast, accurate and comprehensive CNA profiling across a diverse and extensive collection of cancer samples. This achievement serves as a cornerstone for prospective comparative CNA analysis in cancer research.

## MATERIALS AND METHODS

**Figure 1:**
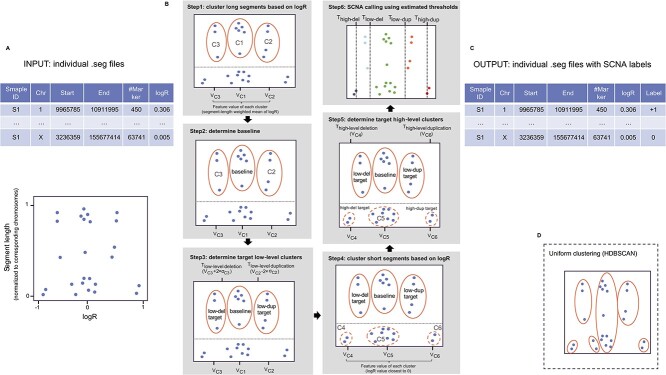
Workflow of labelSeg. (A) Input: The segmented data file provides segment lengths and associated numeric values (logR) for each segment, which are used in subsequent calculations. In the illustration below, each dot represents a single segment, with its normalized segment length displayed on the Y-axis and its logR value displayed on the X-axis. (B) Algorithm: Step 1: Segments are categorized into long and short segments using a cutoff value (0.2) marked by a dashed horizontal line. Long segments are clustered in the logR space (X-axis) using DBSCAN, and these clusters are ranked based on the sum of segment lengths within each cluster. A feature value is computed for each cluster. Step 2: The baseline is determined using the feature value and rank. Step 3: Low-level target clusters are then identified using the distance of the feature value to the baseline and their respective ranks. Step 4: Short segments are also clustered in the logR space using DBSCAN with a larger radius, and these clusters are ranked based on corresponding feature values. Step 5: High-level target clusters are identified considering the distance of the feature value to the baseline, the predefined low-level target clusters and their respective ranks. Calling thresholds are subsequently calculated based on these target clusters. Step 6: The cutoff determined by these estimated thresholds is applied to the segments. (C) Output: The original segment data file is augmented with an additional column of SCNA labels, representing relative copy number states. (D) Uniform clustering: A specialized clustering strategy employed in labelSeg when the clustering method utilizes HDBSCAN, an extension of DBSCAN.

*labelSeg* combines segment length and $\log $R values to estimate appropriate thresholds for calling different levels of SCNA (Figure 1A). The comprehensive implementation of this algorithm is detailed in Figure 1B. There are several assumptions. First, the majority of detected copy number events are driven by predominant clones. It is a prevalent biological assumption that serves as the foundation for most contemporary algorithms utilized in the detection of SCNAs [19]. Under this assumption, segments of individual profiles form distinct clusters in $\log $R values. These clusters are likely to represent different copy number states. Second, arm-level SCNAs are generally low copy number changes, whereas focal SCNAs can be of very high amplitude. This length–amplitude relationship of SCNA, which has been previously reported [16], allows reliable discrimination of different SCNA levels, including low-level duplication/deletion and high-level duplication/deletion.

Currently, the definitions of CNA magnitudes vary across different studies [20–26]. In general, high-level CNAs involve substantial changes in the copy number of chromosomal segments, with high-level duplication indicating the presence of multiple copies of certain genomic regions, and high-level deletion indicating either the complete loss or substantial reduction of specific regions. In contrast, low-level CNAs involve smaller changes in copy number compared with their high-level counterparts. In this study, we proposed the transformation of absolute copy numbers to relative CNA levels, grounded in a consensus derived from the reviewed studies. Specifically, we define high-level duplication as three or more gains compared with the ploidy, low-level duplication as one to two gains, high-level deletion as the absence of any copies and low-level deletion as partial loss.

### Algorithm

#### Clustering

The segments in a sample are divided into long and short segments based on their length relative to the corresponding chromosomes. The criterion for segment size separation was determined based on the empirical distribution of segments derived from a combined dataset across various tumor types (Supplementary Figure S1). Segments occupying $\ge $20% of a chromosome are considered as long segments, and segments occupying <20% of a chromosome are considered as short segments. Long and short segments are subjected to distinct clustering processes based on their $\log $R values. The rationale underlying this choice is rooted in the intrinsic characteristics of long and short segments. Typically, long segments represent broad CNAs and tend to be derived from more measurement markers. As a consequence, long segments exhibit much lower variance and scales in $\log $R values compared with short segments.

We employ the Density-Based Spatial Clustering of Applications with Noise (DBSCAN) [27] algorithm for clustering. There are two important parameters in DBSCAN: $\epsilon $ and $minPts$. The parameter $minPts$ is the minimum number of points required to form a dense region. Intuitively, this value is set to 1 to enable the formation of clusters even with a single segment (e.g. focal amplification). However, users can customize this parameter, and we conducted a benchmark to explore variations in this parameter, as detailed in the Results section. The parameter $\epsilon $ is the maximum distance between two points for one to be considered in the neighborhood of the other. In our algorithm, we determine $\epsilon $ using a self-adaptive method. Initially, these values are set at predetermined levels (0.05 and 0.1 for long and short segment clustering, respectively). Subsequently, $\epsilon $ is systematically reduced by 0.01 until the standard deviations of $\log $R in all clusters fall below specified thresholds (0.05 and 0.1 for long and short segment clustering, respectively). As a result of this variance control, it is possible for segments sharing the same relative copy number state to form multiple clusters in $\log $R values. While this phenomenon might potentially lead to an underestimation of the genuine $\log $R variance within the same copy number state, this underestimation does not hinder the determination of suitable thresholds. This resilience is attributed to both a variance adjustment (see Supplementary Data Section 1.1) and the utilization of specific ‘target clusters’ in our threshold estimation procedure. These ‘target clusters’ are meticulously identified through ranking methodologies that ensure precise threshold determination (elaborated further in the following sections).

Furthermore, our algorithm accommodates alternative clustering methods: Ordering Points To Identify the Clustering Structure (OPTICS) [28] and Hierarchical Density-Based Spatial Clustering (HDBSCAN) [29]. Both methods are extensions of the DBSCAN framework, simplifying the parameterization by removing the need to choose an appropriate $\epsilon $ value. HDBSCAN is a hierarchical extension for varying $\epsilon $ values and automatically determining the optimal number of clusters. It requires only the minimum cluster size ($minPts$) as input. Given that clustering by segment length intends to adapt to clusters of varying density while HDBSCAN is able to overcome such limitation, uniform clustering across all segments via HDBSCAN is reasonable and becomes a viable choice within our algorithm (Figure 1D). OPTICS replaces $\epsilon $ with an upper limit for the neighborhood size (typically rather high and set to infinity in *labelSeg*), generating an ordered list of data points such that points that are spatially closest become neighbors in the ordering. Although OPTICS does not require the $\epsilon $ parameter, it does require a threshold to identify clusters from the OPTICS ordering. In our implementation, the value of the splitting threshold is set to the same value as the $\epsilon $ parameter in DBSCAN, resulting in the actual output of OPTICS comparable with DBSCAN. It is noted that the effect of the $minPts$ parameter in OPTICS and HDBSCAN is different from its role in DBSCAN, as it not only defines the minimal cluster size but also contributes as a ‘smoothing’ factor that involves density estimates.

#### Calculate feature value for each cluster

Following the clustering process, a feature value, denoted as $V_{c_i}$, is computed for each segment group labeled as cluster $c_i$. Depending on whether the cluster corresponds to long or short segments, the calculation methodology varies. For clusters comprising long segments, the feature value is determined as the weighted mean of $\log $R values from segments within the same cluster. Each of these $\log $R values is assigned a weight based on its corresponding normalized segment length relative to the chromosome where it occupies. This weighted mean is used for calculating thresholds in identifying low-level SCNAs. In short segment clusters, the feature value is the $\log $R value closest to 0 within the cluster, e.g. the minimum value in a cluster of positive $\log $R values or the maximum value in a cluster of negative $\log $R values. These feature values within the short segment clusters are instrumental in the derivation of thresholds for detecting high-level SCNAs.

#### Estimate baseline

The baseline, representing segments with a neutral copy number state, is determined from the long segment clustering results. Specifically, the cluster that exhibits a feature value closest to 0 and occupies $\ge $40% of the total measured genomic region is considered as the baseline cluster, denoted as $c_{\text{baseline}}$. In situations where data noise is pronounced or the profile is over-segmented, it is conceivable that no long segment clusters will span over 40% of the total length. In such instances, a step-wise reduction of the percentage limit, from an initial value of 40 to 20%, by a decrement of 10%, is performed until the baseline cluster is found.

During this step, users can interactively adjust the baseline upwards or downwards. The algorithm will subsequently identify the cluster that most closely aligns with the predefined baseline cluster while satisfying the aforementioned criteria.

#### Estimate low-level calling threshold

The next step is to find clusters that represent low-level SCNA events. To increase tolerance to sub-clone effects, the remaining long segment clusters are ranked in descending order based on the cumulative segment length, denoted by $\{c_1,c_2,...,c_k\}$. The target low-level clusters are determined as follows.

$\qquad\qquad\qquad\qquad c_{\text{low-dup target}} = c_a$ ,where 


(1) \begin{align*}& a = \mathop{\arg\min}_{i \in \{1,...,k\}} V_{c_{\text{baseline}}}+0.15 \le V_{c_i} < V_{c_{\text{baseline}}}+0.7;\end{align*}

$\qquad\qquad\qquad\qquad c_{\text{low-del target}} = c_b$ , where 


(2) \begin{align*}& b = \mathop{\arg\min}_{i \in \{1,...,k\}} V_{c_{\text{baseline}}}-1.5 < V_{c_i} \le V_{c_{\text{baseline}}}-0.15\end{align*}

‘Target cluster’ is the cluster used to estimate calling thresholds. The lower bounds in Equations (1) and (2), set at $\pm $0.15, aim to filter out noise from true SCNAs and can be tuned by users, although default values have been chosen based on empirical experience. The upper bounds are set to avoid calling high-level SCNAs and may also be adjusted based on prior knowledge. The default upper bound for duplication, 0.7, is derived from the theoretical $\log $R value observed when the event of 5-copy gain occurs in 70% of diploid cells in the analyzed tissue. Similarly, the default upper bound for deletion, $-1.5$, is based on the theoretical $\log $R value corresponding to a homozygous deletion event occurring in 70% of diploid cells in the measured tissue. When segment profiles exhibit high quality—usually indicative of high tumor purity and low measurement noise—employing higher bounds has the potential to improve the calling performance (Supplementary Figure S3B). To avoid overfitting, all results adhere to the default bounds.

To ensure calling sensitivity, the standard deviation of $\log $R values from segments within the target cluster is included in the calculation of calling thresholds. Let $\text{T}_{\text{low-level SCNA}}$ represent the calling threshold for low-level SCNA, and $\sigma _{c_i}$ denote the standard deviation of $\log $R in the cluster $c_i$. The calculation proceeds as follows: 


(3) \begin{align*} & \text{T}_{\text{low-level duplication}} = V_{c_{\text{low-dup target}}}-2 \times \sigma_{c_{\text{low-dup target}}}; \end{align*}

(4) \begin{align*} & \text{T}_{\text{low-level deletion}} = V_{c_{\text{low-del target}}}+2 \times \sigma_{c_{\text{low-del target}}} \end{align*}

#### Estimate high-level calling threshold

The process of determining high-level calling thresholds integrates the clustering of short segments, the utilization of previously identified target low-level clusters and the numeric correlation of $\log $R distances among distinct copy number states. An interesting observation arises regarding the $\log $R distances between low-level copy changes and neutral copy changes, as well as between high-level copy changes and neutral copy changes. These $\log $R distances demonstrate a robust association with varying tumor sample purity and ploidy. Further insights into this phenomenon can be found in Supplementary Data Section 1.2 and Supplementary Figure S2. By leveraging this established correlation, as shown in Equations (5) and (6), the algorithm effectively finds a reliable calling threshold within heterogeneous samples to distinguish between high-level duplications/deletions and their low-level counterparts.

Short segment clusters are ranked by feature values in ascending order, denoted as $\{c_1,c_2,...,c_m\}$. The target high-level clusters are determined as follows:

$\qquad\qquad\qquad\qquad\qquad c_{\text{high-dup target}} = c_c$ ,

where


(5) \begin{align*}& V_{c_c}> V_{c_{\text{low-dup target}}} \nonumber\\& s_1 = V_{c_{\text{low-dup target}}} - V_{c_{\text{baseline}}} \nonumber\\& c=\mathop{\arg\min}_{i \in \{1,...,m\}} {\frac{V_{c_i} - V_{c_{\text{baseline}}}}{s_1} \ge 2.2-0.6 \times s_1};\end{align*}

$\qquad\qquad\qquad\qquad\qquad c_{\text{high-del target}} = c_d$ ,

where


(6) \begin{align*}& V_{c_d} < V_{c_{\text{low-del target}}} \nonumber\\& s_2 = V_{c_{\text{baseline}}}-V_{c_{\text{low-del target}}} \nonumber\\& d=\mathop{\arg\max}_{i \in \{1,...,m\}} \frac{V_{c_{\text{baseline}}}-V_{c_i}}{s_2} \ge 2\end{align*}

Given the unique characteristics of high-amplitude focal SCNAs and low-amplitude broad SCNAs, such as their different potential to pinpoint oncogenes and tumor-suppressor genes, the identification of high-level SCNAs is refined by calling only those focal SCNAs with amplitudes surpassing those of broad SCNAs. The calling threshold for high-level SCNAs is calculated as follows. Let $\mathbf{V_L}$ be the set of $\log $R values of all previously defined long segments, 


(7) \begin{align*} & {\text{T}_{\text{high-level duplication}} = max(V_{c_{\text{high-dup target}}}, max(\mathbf{V_L})+0.01)}; \end{align*}

(8) \begin{align*} & {\text{T}_{\text{high-level deletion}} = min(V_{c_{\text{high-del target}}},min(\mathbf{V_L})-0.01)} \end{align*}

#### SCNA Calling

Once the estimation of calling thresholds is complete, the subsequent step is to assign labels indicating relative copy number states or SCNA levels to each segment. The classification is carried out according to the following principles:

- Segments with $\log $R $\le $ T$_\text{high-level del}$ are labeled as ‘-2’, indicating high-level deletion. If the sample is diploid, the high-level deletion is a homozygous deletion.
- Segments with $\log $R > T$_\text{high-level del}$ and $\le $ T$_\text{low-level del}$ are labeled as ‘-1’, meaning low-level deletion.
- Segments with $\log $R $\ge $ T$_\text{low-level dup}$ and < T$_\text{high-level dup}$ are labeled as ‘+1’, meaning low-level duplication.
- Segments with $\log $R $\ge $ T$_\text{high-level dup}$ are labeled as ‘+2’, meaning high-level duplication.
- Segments with $\log $R $\gt\! \text{T}_{\text{low-level del}} \text{ and} \lt\! \text{T}_\text{low-level dup}$ are labeled as ‘0’, meaning no changes in total non-allelic copy numbers.

There are other exceptions. For example, in cases where the segment profile is over-segmented and no long segments are present, or only high-level focal SCNAs occur within a sample, specific strategies for handling these exceptions are elaborated in Supplementary Data Section 1.3.

## RESULTS

### Performance on simulated data

The assessment of *labelSeg*’s performance involved a systematic examination of different clustering methods and variations in the $minPts$ parameter, as depicted in Supplementary Figure S3A. The evaluation was conducted using simulated segment data that encompassed different scenarios, including variations in sample purities and noise levels (refer to Supplementary Data Section 2.1 for data generation details). The results from this benchmark indicated a limited impact of parameter choices on the calling performance. Consequently, our attention here is focused on two key configurations within *labelSeg* that were chosen to be representative: distinct DBSCAN clustering with $minPts$ set to 1 for both short and long segments, and uniform HDBSCAN clustering with $minPts$ set to 10. These selections were made based on their specific advantages. The former configuration offers computational simplicity, while the latter leverages the strengths of HDBSCAN, resulting in a specialized clustering strategy (illustrated in Figure 1D). Importantly, these variations do not affect other crucial steps in the algorithm, including individual feature value computation, cluster ranking and threshold determination for both low- and high-level SCNAs.

**Figure 2:**
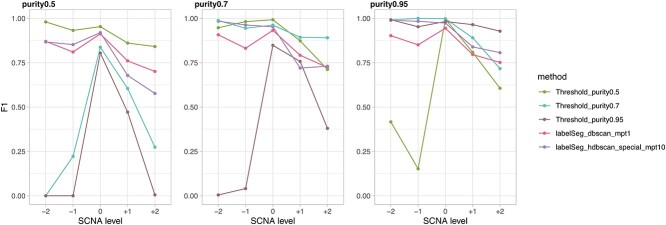
Performance on simulated data. The average F1 scores across samples were calculated for different levels of SCNA calling.

In the simulated scenario with moderate noise, we compared the performance of *labelSeg* with the application of optimal thresholds tailored to specific purity levels (Figure 2). The results were consistent with expectations: optimal thresholds demonstrated superior overall performance when matched with the true tumor purity they were designed for. However, the effectiveness of these thresholds diminished when applied to samples deviating substantially from the target purity. Conversely, *labelSeg* consistently exhibited commendable F1 scores across all simulated scenarios, showcasing its robustness across a broad spectrum of tumor sample purity levels. Notably, the two parameter sets within *labelSeg* showed subtle differences. DBSCAN clustering with $minPts$ set at 1 exhibited enhanced adaptability in scenarios characterized by low sample purity, whereas HDBSCAN uniform clustering with $minPts$ set at 10 excelled in situations of higher sample purity. Even in scenarios with exceptionally high noise levels, all compared methods displayed compromised performance, but this conclusion remained unchanged (Supplementary Figure S3A).

### Performance on real data

**Figure 3:**
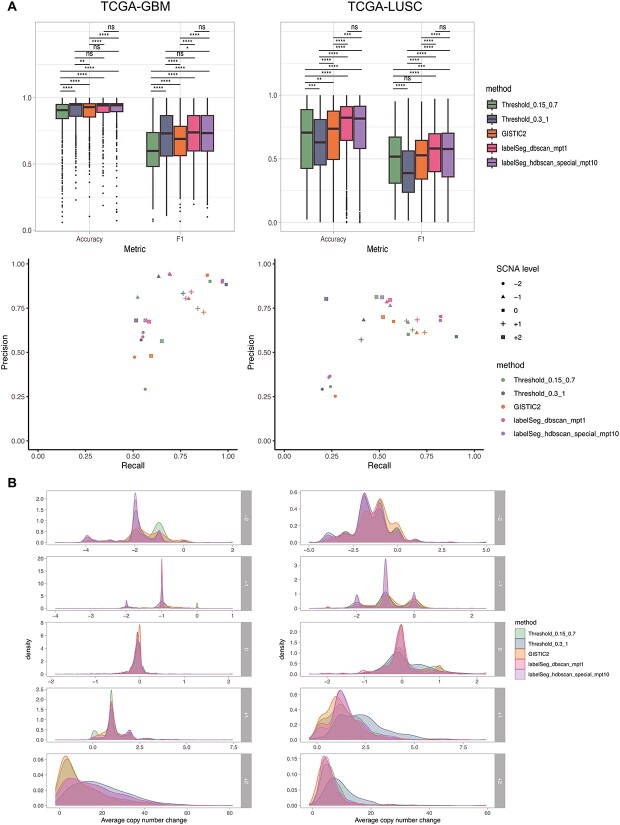
Performance on TCGA datasets. (A) The top panel displays the macro average of F1 scores across different SCNA classes and overall accuracy for each sample. The bottom panel shows the average precision and recall across samples when calling different levels of SCNA. (B) Distribution of absolute copy number change compared with ploidy. For each SCNA level, the average copy change across genes with the corresponding SCNA was considered for each sample.

To evaluate the performance of *labelSeg* on real biological data, we analyzed two TCGA datasets comprising 596 glioblastoma (GBM) and 503 LUSC samples. Both of these tumor types are known to be extensively affected by SCNAs, and these two cohorts have previously been shown to have a difference in average sample purity [30–32]. The evaluation involved a comparison with alternative methodologies, including GISTIC2, a set of more stringent thresholds {$\pm 0.3, \pm 1$} and a set of more relaxed thresholds {$\pm 0.15, \pm 0.7$} (Figure 3). It is worth noting that due to GISTIC2’s gene-level profiling approach for relative copy number states, we conducted a conversion from segment-level labels to gene-level labels for both *labelSeg* outputs and fixed threshold callings. The rationale for excluding GISTIC2 from simulated data validation stems from GISTIC2’s consideration of functional genomic elements in the genome and their varying background rates of SCNAs, a facet not accounted for in the simulation. Consequently, GISTIC2’s applicability was limited in the simulation context. Given the inherent challenge in determining absolute truth within real biological data, we used ASCAT2 [33] estimates as a benchmark reference. These estimates provide gene-level absolute copy numbers extracted from the same datasets, which are then converted into relative CNA levels per gene. While ASCAT2 may not be a gold standard, it benefits from leveraging allelic information, a dimension not included in the benchmarked methods. As a result, the alignment with ASCAT2 calls offers a glimpse into the proficiency of SCNA amplitude profiling. Further details regarding the conversion of SCNA label from segment to gene can be found in Supplementary Data Section 2.2.

In the GBM cohort, *labelSeg* and the stringent threshold (Threshold_0.3_1) had superior performance in terms of F1 score and accuracy (paired Wilcoxon rank sum test) (Figure 3A). Conversely, the relaxed threshold (Threshold_0.15_0.7) had the worst performance, primarily due to compromised precision. This performance discrepancy can also be observed in the distribution of absolute copy number changes for different SCNA levels (Figure 3B). For high-level deletions, *labelSeg* tended to identify CNAs with two or four copies reduction compared with the overall ploidy, suggesting possible complete loss. In contrast, the relaxed threshold was more likely to call CNAs with one copy loss, indicating potential overcalling. Transitioning to the LUSC cohort, *labelSeg* once again demonstrated its prominence in terms of F1 score and accuracy, followed by GISTIC2 and the relaxed threshold. Interestingly, the stringent threshold exhibited limitations in this dataset due to issues of recall, unlike its performance in the GBM dataset. This underscores the inherent challenge posed by fixed thresholds in accommodating diverse tumor purities. As indicated in previous studies, the average sample purity in GBM samples tends to surpass that in LUSC samples [32], which may underlie the performance discrepancy across the two datasets observed in all benchmarked methods. Remarkably, *labelSeg* revealed its strengths in the LUSC dataset by achieving higher assessment scores and producing sharper peaks in the distribution of absolute copy number changes for low-level SCNA calling. These sharp peaks indicate more consistent copy number changes within individual SCNA levels, further highlighting *labelSeg*’s prowess in handling the more complex dataset. Moreover, *labelSeg* showed similar performance across the two representative parameter sets, and additional benchmarking for parameterization can be found in Supplementary Figure S4. In general, when using separate clustering by segment length, it is important to note that large values for $minPts$ can lead to errors due to the limited number of segments in each clustering. Conversely, small $minPts$ values tend to work well with DBSCAN and OPTICS. For HDBSCAN, however, its performance tends to improve when combined with larger values for $minPts$, regardless of whether a uniform or separate clustering strategy is used.

To demonstrate the compatibility of our method across different tumors and platforms, we conducted benchmarking on two additional datasets. We analyzed the acute myeloid leukemia (LAML) dataset from TCGA, which is characterized by a lower frequency of SCNAs in terms of affected sample proportion [34] and we examined a non-TCGA dataset from non-small cell lung cancer samples, generated by MSK-IMPACT targeted sequencing [35, 36]. In the LAML cohort, *labelSeg* and the stringent threshold showed superior performance (Supplementary Figure S5). In the MSK-IMPACT dataset, which exhibits higher noise compared with TCGA data generated by SNP arrays, *labelSeg* displayed a clear advantage when employing separate DBSCAN clustering for both short and long segments with a $minPts$ value of 1 (Supplementary Figure S6).

Taking into account factors such as algorithm complexity and robustness to complicated samples, as demonstrated by the benchmarked results in both simulated and real data, we have designated separate DBSCAN clustering for both short and long segments with a $minPts$ value of 1 as the default parameters for our method.

### Integrative analysis of heterogeneous SCNA profiles

By design, *labelSeg* could provide a reliable calling no matter how heterogeneous the data are in terms of measurement platforms (input is segment file), tumor sample purities and noise (clustering-based estimation). To illustrate its potential for precise and robust SCNA profiling—particularly in discerning between low-level broad SCNAs and high-level focal SCNAs—we applied this method to datasets originating from different resources and performed an integrative analysis. All of the ensuing results are generated using default parameters. There are four data resources from which the data were derived: TCGA, Progenetix [37], Clinical Proteomic Tumor Analysis Consortium (CPTAC) [38] and Cancer Cell Line Encyclopedia (CCLE) [39]. These datasets were generated through various measurement platforms and sample types, with detailed information provided in Table 1 and Supplementary Data Section 3.

**Table 1:** Summary of the glioblastoma segment datasets.

Data resource	Number of samples	Sample type	Platform
TCGA	596	patient tumor samples	Affymetrix SNP 6.0
Progenetix	1390	patient tumor samples and cancer cell lines	CGH array and SNP array
CPTAC	97	patient tumor samples	WGS
CCLE	64	cancer cell lines	WES and WGS

**Figure 4:**
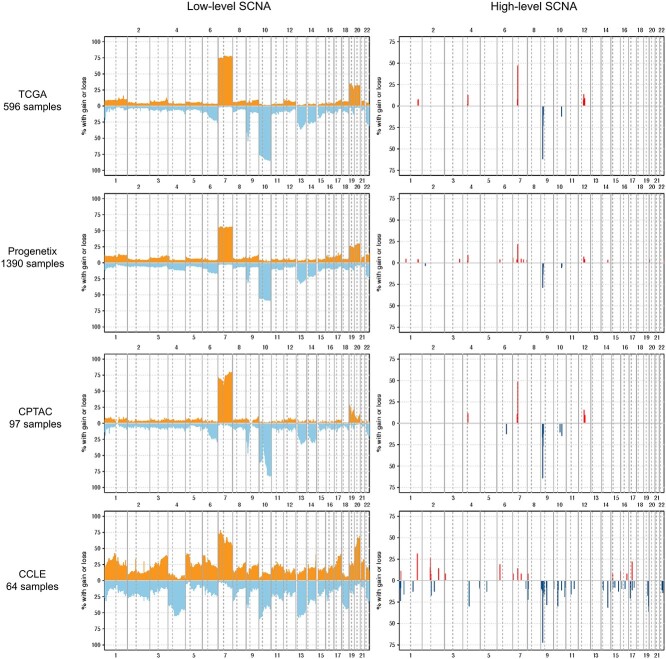
Frequency of SCNA calls in different glioblastoma datasets. The orange and red colors represent duplications, and the light blue and dark blue colors represent deletions. The Y-axis displays the percentage of samples with SCNA overlapping with 1MB-sized genomic bins. The X-axis denotes chromosome numbers. Low-level SCNAs are identified by segments labeled ‘+1’ and ‘-1’, while high-level SCNAs are identified by segments labeled ‘+2’ and ‘-2’. In the frequency plots of high-level SCNAs, background noise peaks were filtered out.

In glioblastoma samples, the SCNA patterns from different datasets were relatively consistent (Figure 4). Low-level SCNA frequency was calculated from segments with labels ‘+1’ and ‘-1’. Chromosome 7 duplication and chromosome 10 deletion were the most prominent low-level SCNA features in glioblastoma samples with occurrence greater than 50% in all datasets and reaching 75% in the TCGA and CPTAC datasets. Chromosomes 9p, 13, 14 deletions and chromosomes 19, 20 duplications occurred in more than 25% of samples in most datasets. The UMAP plots based on the called low-level SCNA coverage of individual samples (Supplementary Figure S7) reveal the association of SCNAs across the genome. The co-occurrence of chromosome 7 duplication and chromosome 10 deletion was frequent in the analyzed samples. Duplications of chromosomes 19 and 20, and deletions of chromosome 9p were more likely to occur in samples with simultaneous chromosome 7 duplication and chromosome 10 deletion (*P*-value < 3e-12 for chr19, *P*-value < 9e-06 for chr20, *P*-value < 0.003 for chr9p, Pearson’s Chi-squared test). The SCNA pattern observed in the CCLE dataset was characterized by greater heterogeneity and noise compared with other datasets. This could possibly be explained by the difference between tumor samples and cell line models [40].

High-level SCNA frequency was calculated from segments with labels ‘+2’ and ‘-2’. Multiple consensus high-level SCNA focal peaks were observed across various analyzed projects, indicating the recurrent SCNAs’ robustness and reliability. Similar to low-level SCNA pattern, the CCLE samples were also more heterogeneous in high-level SCNAs. Supplementary Tables S1–S2 provide further information regarding congruent high-level duplication peaks from at least two projects and high-level deletion peaks from at least three projects. The most frequent amplification cross datasets happened in chr7: 54.1–56.1 MB with a frequency of around 46% in TCGA and CPTAC, 21% in Progenetix and 12% in CCLE. The most frequent high-level deletion (probably homozygous deletion) occurred in chr9: 21–23 MB with a frequency around 62% in TCGA and CPTAC, 28% in Progenetix and 70% in CCLE. The reduced high-level frequency detected in the Progenetix samples could potentially be attributed to the heterogeneity of the microarrays employed, which vary in their detection sensitivity of small CNAs, in conjunction with the diversity of sample types analyzed (Supplementary Figure S8). Amplification peaks were identified at chr6: 31.8–32.8 MB and chr7: 92.1–93.1 MB exclusively in the CCLE and Progenetix cohorts. Since all analyzed CCLE samples are cell lines, we hypothesized that amplification in these peaks is more likely to occur in glioblastoma cell lines. To test this hypothesis, we examined the composition of the Progenetix samples that exhibited such amplification. We found that a considerable proportion of the samples (37.6%) with the interesting amplification were cell lines, which is significantly higher than the overall proportion of cell lines in the Progenetix samples (6.3%). This observation was confirmed by a Pearson’s Chi-squared test with a *P*-value less than 2.2e-16, indicating a potential association between cell line samples and amplification in those loci.

We also analyzed the LUSC datasets from these data resources (Supplementary Figure S9). Low-level and high-level SCNA patterns in LUSC samples were roughly accordant across projects. Duplications of chromosomes 1q, 2p, 3q, 5p, 7, 8q and deletions of chromosomes 3p, 5q, 8p were characteristic low-level SCNA patterns in these samples with frequencies between 25 and 50%. Apart from focal peaks, high-level SCNAs spanned chromosomes 3q and 5p with amplification.

### Relationship between copy-number dosage and messenger RNA expression

**Figure 5:**
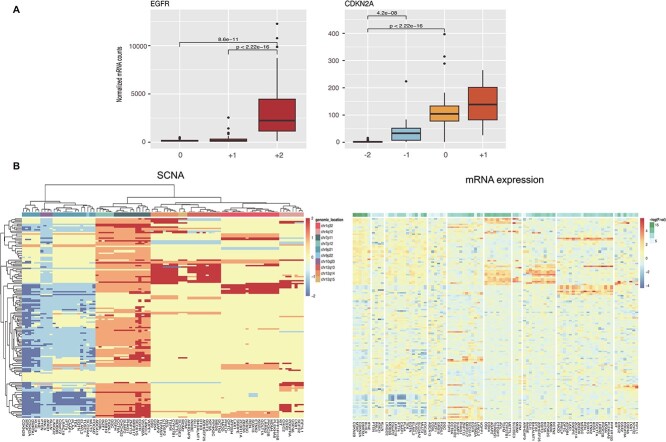
mRNA expression of genes with frequent high-level duplications and high-level deletions in glioblastoma. (A) mRNA expression of characteristic genes across different SCNA levels. (B) Heatmap displaying SCNA levels and mRNA expression in TCGA glioblastoma samples. The rows correspond to samples, and the columns correspond to genes. The left SCNA heatmap presents SCNA label values. The right mRNA heatmap maintains the same row and column order as the left plot. *P*-values were computed using the Kruskal–Wallis rank sum test and BH-adjusted from TMM-normalized mRNA counts. These normalized mRNA counts were log-transformed and standardized across samples per gene for visualization.

SCNAs have been reported to exhibit a strong correlation with messenger RNA (mRNA) expression. However, it is important to note that these alterations do not lead to proportional changes in gene expression levels, due to the presence of transcriptional adaptive mechanisms [41, 42]. To better understand the extent to which SCNAs influence the expression of specific genes and to demonstrate the practical utility of *labelSeg* and the information provided by CNA levels, we examined the correlation between copy-number dosage and mRNA expression of protein-coding genes with recurrent high-level CNAs. Specifically, we analyzed paired mRNA expression data (normalized STAR read counts by TMM [43]) and CNA profiles of glioblastoma samples from the TCGA-GBM project. We only considered protein-coding genes that were amplified or highly deleted with a frequency of >5% in TCGA-GBM samples, and that were located in the consensus focal regions mentioned in Section 4.3 and Supplementary Tables S1–S2. Our results showed that 62 out of 68 frequently amplified genes had significantly increased mRNA expression when the SCNA level was ‘+2’ compared with those with copy-neutral SCNAs labeled as ‘0’ (Kruskal–Wallis test). Similarly, 21 out of 24 frequently high-level deleted genes had significantly decreased mRNA expression when the SCNA level was ‘-2’ compared with those with SCNAs labeled as ‘0’. In this cohort, the most commonly observed high-level duplicated gene was the tumor oncogene EGFR, while the tumor suppressor gene CDKN2A was the most frequently deleted. We found a robust association between the levels of SCNAs and the corresponding mRNA expression for both of these genes (Figure 5A). Supplementary Figures S10–S11 provide box plots illustrating the correlation between SCNA and mRNA expression for other genes.

Figure 5B further shows the high correlation between SCNA levels and mRNA expression of the frequently altered genes in copy number dosage. These genes were clustered based on their genomic location in SCNA levels, which is not surprising since SCNA is a large-scale genomic variation affecting multiple genes. Genes in cytobands chr7p11, Chr9p21, chr12q13 and chr12q14 were strongly regulated in mRNA expression by the relative copy number states. Interestingly, VSTM2A and ARHGAP9 were not influenced by SCNA levels, although mRNA expression of nearby genes was strongly impacted by SCNA. Enrichment analysis was performed separately for these frequently high-level duplicated and deleted genes (Supplementary Figure S12). Both sets of genes were enriched in the glioma signaling pathway, indicating the important role of high-level SCNA in cancer development.

A similar analysis was conducted on the TCGA-LUSC cohort. Of the 1383 frequently amplified genes, 938 had an associated mRNA expression with their SCNA levels, while 20 of the 25 frequently high-level deleted genes had an associated mRNA expression with their SCNA levels. Notably, the high-level deleted genes with mRNA expression responsible for copy number changes were also located in cytoband chr9p21, which contains CDKN2A and CDKN2B (Supplementary Figure S13). Several frequently amplified genes that did not show mRNA association were enriched in epidermal keratinocyte differentiation (Supplementary Figure S14).

## CONCLUSION

Comprehensive profiling of SCNAs is valuable for advancing our understanding of cancer development and improving precision medicine applications. Here, we provide a novel method *labelSeg* for fast and accurate annotation of CNA segments. Our method estimates thresholds for calling different CNA levels from individual segment profiles, allowing more complete and robust identification of SCNAs in heterogeneous samples. The use of separate clustering by segment length not only adapts to the biological and technical variance in both focal and broad segments but also increases the tolerance to sub-clone effects and noise. Compared with the use of fixed cut-off values, *labelSeg* achieves a similar calling speed but increased accuracy. Furthermore, it does not require additional estimation such as tumor sample purity or other prior knowledge, making it a more convenient and powerful tool for large-scale comparative and integrative analysis to overcome bias from individual studies or platforms.

Our study demonstrated that *labelSeg* outperformed previous methods in SCNA profiling, as evidenced by its higher accuracy and F1 score. The robustness of *labelSeg* was demonstrated by the consistent patterns of SCNAs detected across heterogeneous datasets, making it a suitable tool for integrative analyses. Our analysis further confirmed simultaneous chromosome 7 duplication and chromosome 10 deletion in glioblastoma samples, which has been previously reported in other studies [31], thus highlighting the detection accuracy of genome-wide low-level CNAs. Furthermore, we identified several consensus high-level SCNA focal peaks enriched in protein-coding genes, which were observed in at least two of the four datasets. For most of these genes, mRNA expression strongly correlated with the called SCNA status, providing insights into the impact of SCNA of driver genes on tumor evolution.

Because *labelSeg* solely requires the $\log $R values of segments as input, it is compatible with any technology that delivers segment data from its processing pipeline, regardless of the original data type (i.e. count or intensity-based). However, this general compatibility to a wide range of e.g. microarray and NGS platforms arrives with certain limitations of the method, particularly in estimating absolute copy number and ploidy which require some information about allelic composition. Although this is not a problem when relative copy number states are targeted, it renders the method unsuitable for some investigations that require absolute quantification of copy numbers such as the accurate assessment of aneuploidy in tumor cells. Also, while sensitivity to probe-level noise affects all calling methods to various degrees, in *labelSeg* such noise can diminish the clustering of segments in the $\log $R values, which can hinder the ability to set accurate calling levels. Therefore, when applying *labelSeg* to segment data generated by platforms with higher noise levels, such as methylation arrays and single-cell RNA sequencing, its performance might be compromised. However, when working with segment data of good quality, these limitations become less concerning.

To conclude, our study presents a new strategy for segment classification and annotation, which enhances the interpretation of heterogeneous segment profiles with respect to calling efficiency, accuracy and granularity. The stratification of SCNAs based on distinct levels highlights their varying sizes, amplitudes and potential functional roles in the context of cancer pathogenesis. As the role of SCNA information expands within cancer genomics applications, including tumor classification and clinical diagnostics[44–46], our dedicated profiling method not only empowers us to investigate the intricate relationship between SCNAs, tumor evolution and oncogenomic subtypes but also serves as a valuable tool for precise patient profiling. By identifying specific SCNAs associated with prognosis and treatment response, our method has the capacity to assist clinicians in tailoring therapeutic strategies, thereby advancing the field of precision medicine and ultimately contributing both to advancements in cancer research and clinical care.

**Key Points**

- Somatic copy number alterations (SCNAs) in cancer genomes play a crucial role in tumor initiation and progression. Currently, the interpretation of segment data, a common data format for representing SCNAs, is challenging due to signal variability and noise.
- To address this challenge, we present a novel method called *labelSeg*. This tool employs the Density-Based Spatial Clustering of Applications with Noise (DBSCAN) clustering algorithm and leverages the length–amplitude relationship of SCNAs to identify distinct CNA types from individual segment profiles, including low-level broad CNAs and high-level focal CNAs.
- *labelSeg* showed superior performance over alternative methods in both simulations and real datasets. And the usefulness of *labelSeg* was illustrated in the integrative analysis of different segment datasets and in the analysis of tumor key genes targeted by frequent high-level SCNA occurrences.

## Supplementary Material

Supplementary_data_bbad541

## Data Availability

The *labelSeg* R package is available on GitHub at https://github.com/baudisgroup/labelSeg. The average execution time under default parameters was approximately 0.005 s per sample with 200 segments on a MacBook Pro18,4 (Apple M1 Max, 10 cores, 64 GB RAM) using 1 core.

## Author Biographies

**Hangjia Zhao** is a PhD student in the Department of Molecular Life Sciences at the University of Zurich.

**Michael Baudis** is a Professor in the Department of Molecular Life Sciences at the University of Zurich.
